# Supplementary material for: The genome sequence of Hornet Plumehorn hoverfly, Volucella zonaria (Poda, 1761)
Source: Wellcome Open Res. 2025 Feb 19;10:86. [Version 1] doi: 10.12688/wellcomeopenres.23738.1 (PMC12134739; doi:10.12688/wellcomeopenres.23738.1)
Supplement: Peer review history

# Reviewer report: Reviewer response for version 1

**DOI:** 10.21956/wellcomeopenres.26186.r119131

**Published:** 2025-06-03

**Authors:** Jonathan Wilson, Nina Wedell

**Affiliations:** 1 The University of Melbourne, Melbourne, Victoria, Australia; 2 School of BioSciences, The University of Melbourne Faculty of Science (Ringgold ID: 98487), Melbourne, Victoria, Australia

Wolton *et al.* have reported a well assembled chromosome-level reference genome for *Volucella zonaria*. This assembly captures the majority of the genome within 5 autosomes and 2 sex chromosomes. They have summarised the results of the genome assembly nicely, and there are no obvious issues with any methods or findings that could detrimentally affect subsequent genomic analyses involving *V. zonaria*. I have some minor comments about metrics to report, and possible additional analyses that may be performed in future improvements to this genome assembly.

  

1. If there are any previous estimates of chromosome number available, it would be worth mentioning them (though the 7 pseudomolecules seem very likely to represent the true chromosome number). It would also help to state the genome size estimate (from GenomeScope2) explicitly in text – is it the 1262.78 Mb in Table 2?
2. The BUSCO results you have reported look adequate, however the similarity between the BUSCO completeness percentage (90.6%) and the percentage of assembly mapped to the chromosomes is interesting. Have you run the BUSCO analysis exclusively on the chromosomes to see how many BUSCOs are on the chromosomes? While not of huge importance, this might provide a better picture of how complete each chromosome is and could be important information for downstream analyses. Also, it is possible that some missing BUSCOs may lie within the alternate contigs. Including the alternate assembly in a subsequent BUSCO run could indicate whether some genes are missing only on the primary haplotype.
3. Finally, in future it would be worth further investigating the alternate assembly. It would be helpful to report metrics from the alternate haplotype, especially given the k-mer completeness appears to be higher in the alternate assembly (Table 2). Also, aligning the alternate contigs to the primary assembly would be interesting. This could help in efforts to understand structural variation within this species and future efforts to create a pangenome for this species and/or its relatives and may also resolve some of the missing BUSCOs.

Are sufficient details of methods and materials provided to allow replication by others?

Yes

Is the rationale for creating the dataset(s) clearly described?

Yes

Are the datasets clearly presented in a useable and accessible format?

Yes

Are the protocols appropriate and is the work technically sound?

Yes

Reviewer Expertise:

evolutionary biology, bioinformatics, genomics, population genetics

We confirm that we have read this submission and believe that we have an appropriate level of expertise to confirm that it is of an acceptable scientific standard.

# Reviewer report: Reviewer response for version 1

**DOI:** 10.21956/wellcomeopenres.26186.r119132

**Published:** 2025-02-26

**Authors:** Bhagya C. Thimmappa

**Affiliations:** 1 USDA Agricultural Research Service, Chatsworth, NJ, USA

In this work, the authors report a chromosomal-level assembly of the Hornet Plumehorn hoverfly, *Volucella zonaria *(Poda, 1761). The report is concise and well-written, with a sufficient introduction and a detailed method section. The nuclear and mitochondrial genome assemblies are of good quality, and with manual curation, the authors improved the assembly by correcting 11 misjoins or missing joins and removing one haplotypic duplication.

 Comments:

- The annotation of the nuclear and mitochondrial genomes would further increase the value of these datasets.
- It is mentioned that the nuclear genome will be annotated using RNA-Seq data. When is this expected to be completed? How will the annotated genome be made accessible once it is finished? How many RNA-Seq datasets will be used, and where are these datasets from? Are they publicly available?

Are sufficient details of methods and materials provided to allow replication by others?

Yes

Is the rationale for creating the dataset(s) clearly described?

Yes

Are the datasets clearly presented in a useable and accessible format?

Yes

Are the protocols appropriate and is the work technically sound?

Yes

Reviewer Expertise:

Genomics, Trasnscriptomics, Bioinformatics, Multi-Omics analysis

I confirm that I have read this submission and believe that I have an appropriate level of expertise to confirm that it is of an acceptable scientific standard.
